# Significances of viable synergistic autophagy-associated cathepsin B and cathepsin D (CTSB/CTSD) as potential biomarkers for sudden cardiac death

**DOI:** 10.1186/s12872-021-02040-3

**Published:** 2021-05-08

**Authors:** Jialin Dai, Qiong Zhang, Changwu Wan, Jiangjin Liu, Qiaojun Zhang, Yanni Yu, Jie Wang

**Affiliations:** School of Forensic Medicine, Guizhou Medical University, 4 Beijing Road, Guiyang, 550001 Guizhou China

**Keywords:** CTSB/CTSD, Biomarker, Expression, Autophagy, Sudden cardiac death

## Abstract

**Background:**

The Cathepsins family, including cathepsin B and cathepsin D, potentially affects the entire processes involved in atherosclerosis. Although coronary heart disease (CHD) has been widely studied as the basis of Sudden Cardiac Death (SCD), the relationship between CHD and CTSB/D remains unclear.

**Methods:**

We screened for differentially expressed proteins (DEPs) associated with autophagy by limma package in R. For the genes corresponding to the DEPs after screening, we used various databases to carry out functional enrichment of related DEGs to explore their possible influence on a specific aspect of the disease. Functional enrichment analysis of DEGs was performed by DAVID, Metascape and GSEA. STRING and Cytoscape were obtained the hub genes, the analysis of interaction networks through the GENMANIA and Networkanalyst. Western Blot was used to validate the protein expression level of target genes. TF and miRNA prediction were performed using Networkanalyst and visualized using Cytoscape.

**Results:**

The expression levels of members of the cathepsin family were up regulated in CHD tissues compared with the control. GO and KEGG revealed that cathepsin was markedly enriched in endopeptidase activities, immune responses, lysosome pathways, et al. The correlation analysis showed that in patients with CHD, the CTSB/CTSD expression were negatively correlated with ATG4D and BNIP3, but positively with BCL2L1, CAPNS1, and TP53. In the TF-mRNA-miRNA network, has-miR-24-3p and has-miR-128-3p had higher degrees, CTSB/CTSD could be targeted by them.

**Conclusions:**

Our findings elucidated the expression and regulatory role of cathepsins in coronary heart disease induced SCD and might further explore the potential mechanisms of autophagy in CHD.

## Introduction

Sudden cardiac death (SCD) is an unexpected death caused by the sudden cessation of cardiac activity. It is the most significant cause of natural death in the world, accounting for 170,000 to 450,000 adult deaths in western countries, including the USA, and 544,000 adult deaths in China annually [1, 2]. The pathogenesis of SCD is extremely complex, coronary heart disease (CHD) is certainly the most common disease contributing to SCD [3]. Despite discovery of risk factors, innovative diagnostic modalities, therapeutic interventions, and new drug targets development, have led to mortality rate reduction, but the incidence of SCD as a proportion of overall cardiovascular death has remained relatively constant [4]. Therefore, identifying accurate molecular markers is very important for SCD diagnosis and treatment.

Autophagy is an essential process of the catabolic mechanism, which promotes cell survival by eliminating damaged or defective organelles and releasing energy substrates via the degradation of cellular constituents. However, an uncontrolled and excessive autophagic activation can trigger cell death via the depletion of essential organelles and molecules [5]. Because autophagy can eliminate misfolded proteins and damaged organelles [6], and supply substrates for ATP regeneration during ischemia and starvation [7, 8], these functions can maintain cardiac structure and function. So, autophagy is considered as essential for the maintenance of cardiovascular homeostasis and function [9]. Coronary obstruction and microcirculation disorder lead to myocardial ischemia and hypoxia in the pathogenesis of CHD, autophagy was activated, it could be emove damaged organelles, and inhibit inflammation, leading to anti-inflammatory effects and stabilization of atherosclerotic plaques. The process is considered an adaptive response with cardioprotective effects [10–12]. On the contrary, studies have shown that if autophagy becomes dysfunctional, which stimulates hyperactivation of inflammasomes to promote atherogenesis [13].

Cathepsins are the primary lysosomal proteases, they are naturally expressed in the lysosomes of various cells and tissues. Cathepsins contribute to arterial plaque formation and underlie clinical events by extracellular matrix digestion, thereby rendering plaques prone to rupture. In addition, lysosomes also mediate a variety of homeostatic processes such as nutrient breakdown and removal of damaged organelles [14, 15]. Cathepsins are subdivided into 3 subfamilies based on the active-site amino acids: Serine cathepsin (CTSA/G), aspartic cathepsin (CTSD/E), cysteine cathepsin (CTSB/C/F/H/K/L/O/S/V/W/Z) [6]. CTSB and CTSD were extracted as the autophagy-related genes from Human Autophagy Database [16] (HADb, http://www.autophagy.lu/index.html). Studies conducted in vitro or in vivo models have revealed that over-expression of CTSB/D can contribute to the formation of arterial plaques and increase the risk of coronary artery disease. However, the relationship between CTSB/D and coronary heart disease in autophagy regulation remains unclear. It is necessary to have a comprehensive and in-depth understanding of the role of autophagy of the above two genes in the process of SCD.

We studied CTSB/D expression in data from coronary arteries of SCD in private protein datasets. Moreover, we analyzed genomic alterations and functional networks related to autophagy-related proteins CTSB/D to determine their expression patterns, the potential functions, and the correlation between autophagy and risk factors and autophagy pathways. Thus, our results could potentially reveal new autophagy-related targets and strategies for SCD diagnosis and treatment.

## Methods

### Microarray data and identification of differentially expressed proteins (DEPs)

Given the small number of datasets for the human Coronary artery in SCD. For this study we selected three data sets for analysis. The one dataset was obtained from Label-free quantitative proteomics which contained 6 samples, including 3 non-coronary atherosclerosis tissues samples and 3 coronary atherosclerosis tissues of SCD. Principal component analysis (PCA), a commonly method for sample clustering, was used to test the intra-group dataset repeatability. In the following study, we identified some DEPs by using Limma from R (Version:4.0), the values in which statistical significance applies were set to P-values < 0.05 and |log2 fold change (FC)|≥ 1. Volcano maps were drawn using the imageGP (http://www.ehbio.com/ImageGP/) online analysis tool. The other two datasets were GSE12288 and GSE20680 from the GEO database, where GSE12288 was a sample of 222 patients with 110 coronary artery disease (CAD) and 112 without CAD; GSE20680 was a sample of 195 patients with > 1 large vessel stenosis ≥ 70% or > 2 arterial stenoses ≥ 50%, patients with luminal stenosis > 25% but < 50% and controls with luminal stenosis ≤ 25%. The above two datasets were primarily used for subsequent autophagy-related protein correlation analysis.

### KEGG and GO enrichment pathway analyses of differentially expressed genes(DEGs)by Metascape and DAVID

For the genes corresponding to the *differentially expressed proteins (*DEPs) after screening, we used various databases to carry out functional enrichment of related *differentially expressed genes (*DEGs) to explore their possible influence on a specific aspect of the disease. DAVID, (an online analysis tool (https://david.ncifcrf.gov), used to provide biofunctional comprehensive and systematic annotation information for a list of large-scale proteins or genes), was used to perform KEGG and GO annotations pathway analysis of DEGs. It is mainly used for the enrichment analysis of the function and pathway of the genes of DEGs [17].The enrichment analysis was visualized by using ggplot2 from R. Then we used the Metascape database (https://metascape.org/gp/index.html) for GO enrichment analysis and KEGG pathway enrichment analysis again [18]. A P-value < 0.05 was considered statistically significant.

### Gene set enrichment analysis (GSEA)

Identifying the prospective function for genes via the GSEA software [19], one can get a comprehensive biological functional understanding for that gene, in particular, via the enrichment of function sets. To ascertain if prior biological processes in the gene rank derived from DEGs between both groups were enriched, GSEA was conducted (http://software.broadinstitute.org/gsea/index.jsp) [20]. A false discovery rate (FDR) < 0.25 and a P-value of < 0.05 were considered to be statistically significant, while the number of permutations was set at 1000.

### PPI network analysis and extraction of hub genes

We imputed the gene symbol of the DEGs into the STRING (https://string-db.org/) (version 11.0), to evaluate the interactive relationships among the 148 DEGs. Medium confidence of > 0.4 and a minimum required interaction score were deemed significant. Cytoscape software [21] (version 3.4.0, http://chianti.ucsd.edu/cytoscape-3.4.0/) was then used to construct PPI networks, while plug-in molecular complex detection (MCODE) was used to screen PPI network modules in Cytoscape. Hub genes were excavated based on a combined score of ≥ 10. In addition, analysis of gene–gene interaction networks through the GENMANIA online analysis tool and Clustering analysis of hub genes using Networkanalyst. (https://www.networkanalyst.ca/) [22].

### Western blot analysis

Coronary artery tissues were frozen in liquid nitrogen. Total protein was extracted from Coronary artery using RIPA lysis buffer (P0013B, Beyotime) with PMSF (ST506, Beyotime), resolved by 12% sodium dodecyl sulfate polyacrylamide gel electrophoresis (SDS-PAGE) and transferred onto polyvinylidene fluoride (PVDF) membranes by electroblotting. CTSB/C/D/Z proteins were detected using monoclonal antibody, then they were subjected to horseradish peroxidase (HRP) -labeled goat anti-rabbit IgG polymer (1:5000). After the addition of developer and post-exposure, grayscale values were measured using ImageJ analysis software (National Institutes of Health, USA) and internal controls were used with β-actin.

### Screening and correlation analysis of autophagy-related genes

Screening cathepsin from the available datasets, genes in GSE12288 and GSE20680 were compared to 222 autophagy genes in the Human Autophagy Database (http://www.autophagy.lu/). Then we analyzed the presence of a linear correlation between cathepsin and autophagy-related genes in other data sets.

### Predictive studies of TF-mRNA-miRNA regulatory networks

Firstly, the mRNA-miRNA and the TF-mRNA interactions were predicted with Networkanalyst online tools. We next extracted the miRNAs that were predicted to interact with both TF and mRNAs. The TF-mRNA-miRNA regulatory network was constructed from these miRNAs and their targets, while the final network was visualized by Cytoscape. P-value < 0.05 was considered statistically significant.

## Results

### Validation of the datasets and identification of DEGs

**Fig. 1:**
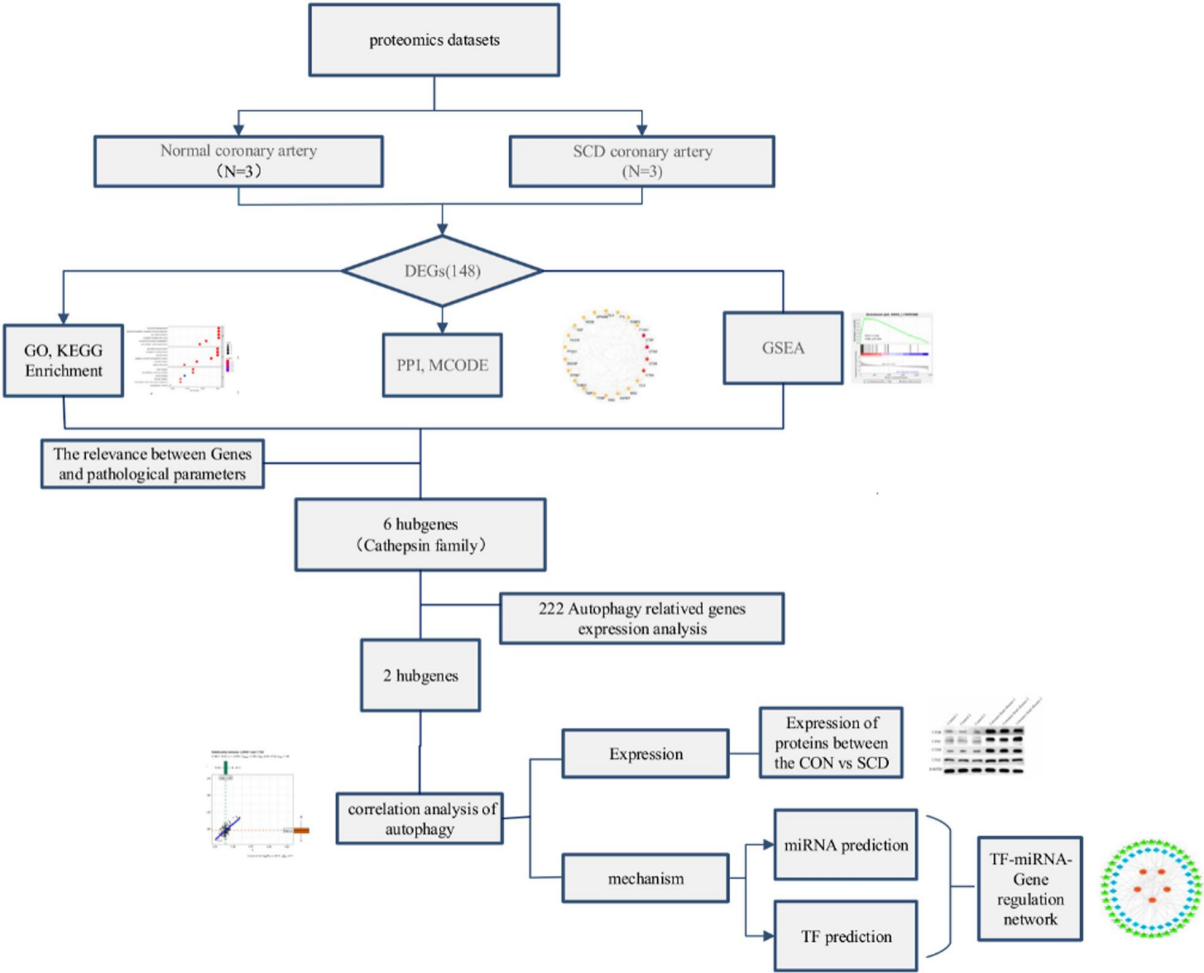
A flowchart of data analysis

**Fig. 2:**
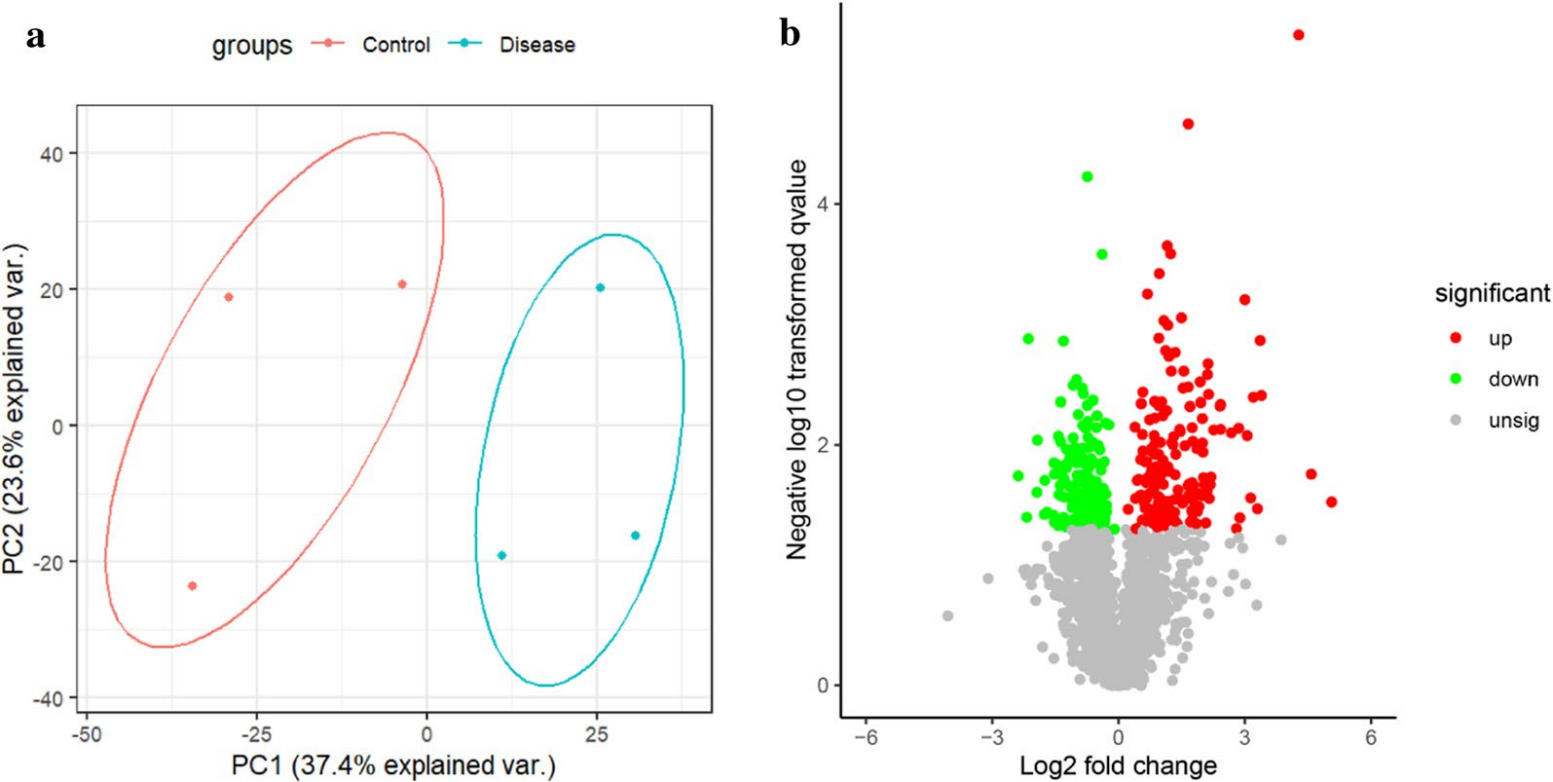
**a** The quantitative principal component analysis results of 6 samples were shown in the figure. The higher the degree of aggregation between repeated samples, the better the quantitative repeatability. **b** The volcano plots showed the genetic differences between the normal and coronary heart disease groups

In this study, the specific workflow is shown in Fig. 1. We employed the PCA to substantiate the intra-group data repeatability. Based on the PCA the intra-group data repeatability for protein dataset was acceptable. The distances between per samples in the control group were close and the distances between per samples in the CHD group were also close in the dimension of principal component-1 (PC1) (Fig. 2a).

After analyzing the datasets with the Linear Models for Microarray (LIMMA; Version:4.0) affy in R package, we used P-value < 0.05 and |log2 FC|> 1 as the cut-off criteria. We extracted 148 DEPs from our datasets, including 101 up-regulated DEPs and 47 downregulated DEPs. The screened DEPs were plotted as volcano plots between control and SCD samples (Fig. 2b). Therefore, the related DEG can be used for subsequent analysis.

### Functional enrichment of DEGs

**Fig. 3:**
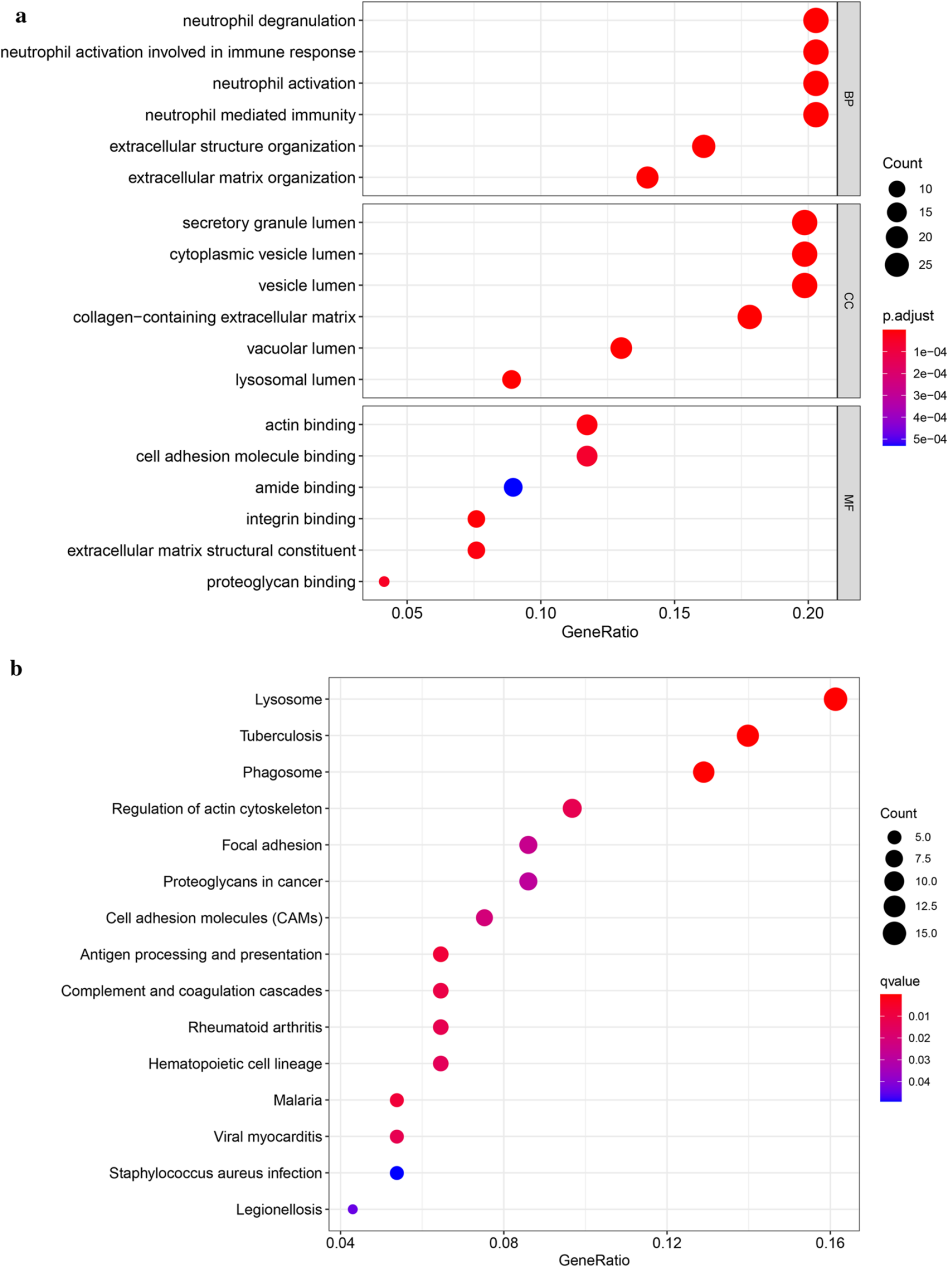

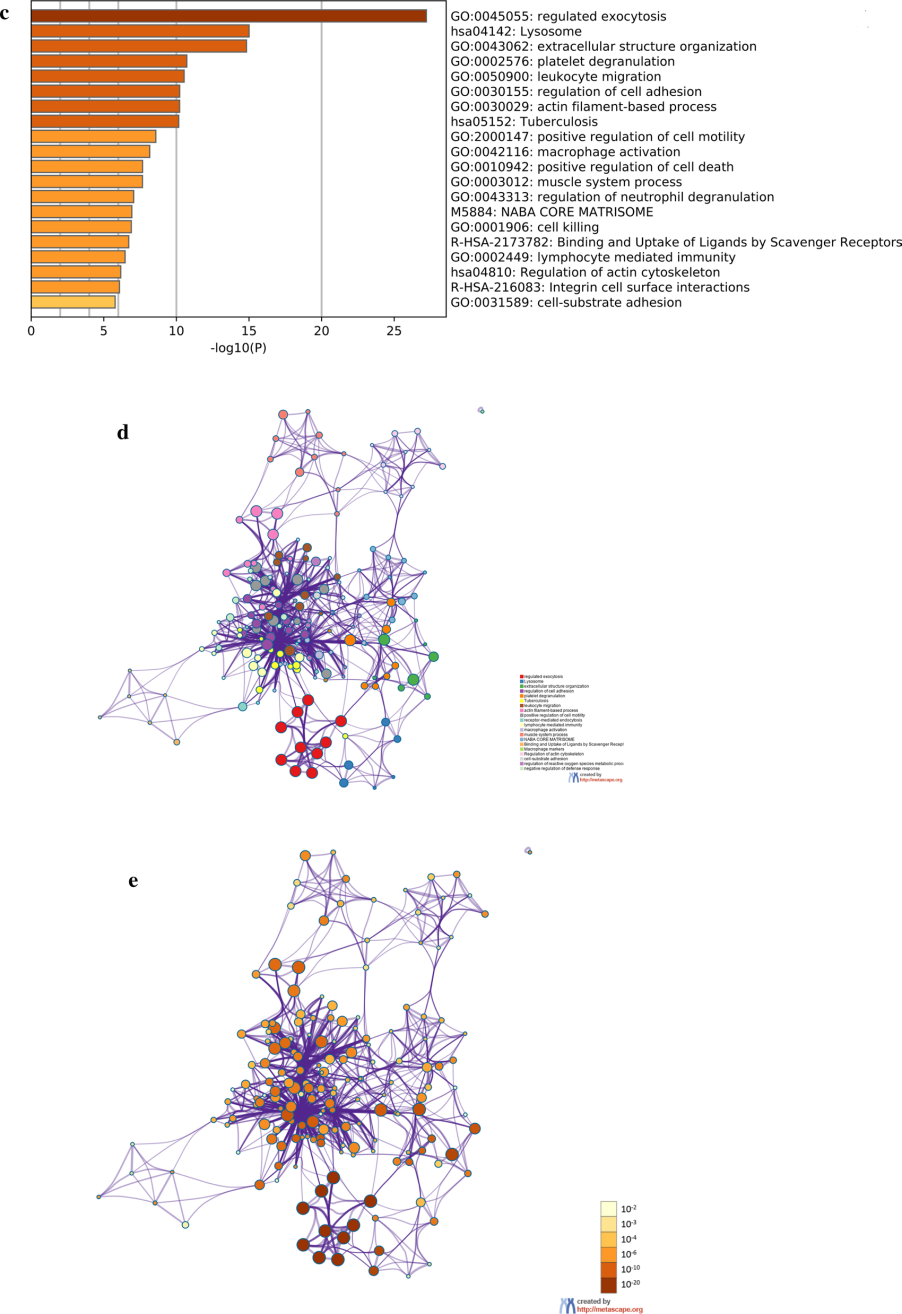

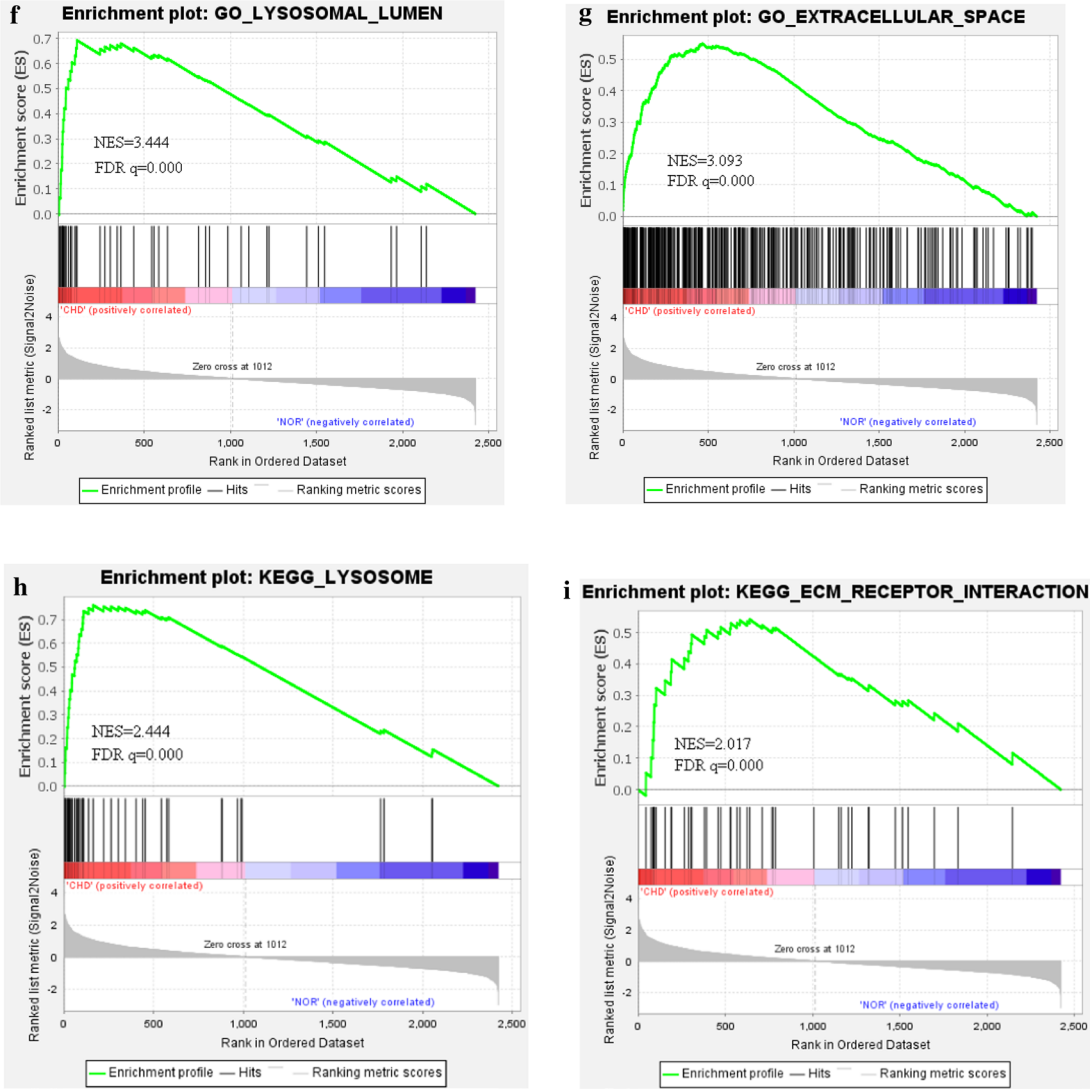
**a** Detailed information relating to changes in the biological processes (BP), cellular components (CC), and molecular functions (MF) of DEGs in Coronary heart disease and control tissues through the GO enrichment analyses. **b** The KEGG pathway analysis of up-regulated or down-regulated DEGs. **c** Heatmap of enriched terms via the Metascape. **d** Network of enriched terms from each cluster is selected to have its term description shown as label. **e** Network of enriched terms has its nodes colored by p-value, as shown in the legend. The darker the color, the more statistically significant the node is (see legend for p-value ranges). **f**, **g** Functional enrichment analysis of DEGs in SCD using GSEA. **h**, **i** Pathway enrichment analysis of DEGs in SCD using GSEA

In order to gain insight into the function of identified related DEGs in SCD, the results of the GO analysis revealed that there were markedly enriched in biological processes (BP), including neutrophil degranulation, neutrophil activation involved in immune response. The variations in cell components (CC) of DEGs were markedly enriched in the secretory granule lumen, lysosomal lumen and collagen-containing extracellular matrix. The variations in molecular function (MF) were markedly enriched in endopeptidase activity, actin binding and cell adhesion molecule binding (Fig. 3a). Moreover, analysis of the KEGG pathway revealed that the DEGs were primarily enriched in Lysosome, Phagosome, Regulation of actin cytoskeleton (Fig. 3b). Metascape online analysis software was used to analyze the DEGs, and the similar enrichment results were obtained as that of DAVID software (Fig. 3c–e).

The biological significance of more genes in the datasets were analyzed by GSEA. Significant GO term analysis by GSEA showed that 906/1721 gene sets were up-regulated in SCD, 523 gene sets were significantly enriched at nominal P-value < 0.05. In addition, 815/1721 gene sets are downregulated in SCD, 433 gene sets are significantly enriched at nominal P-value < 0.05. GSEA also revealed that up-regulated gene sets in SCD were mainly associated with lysosomal lumen (NES = 3.444, FDR q = 0.000), and extracellular space (NES = 3.093, FDR q = 0.000) (Fig. 3f, g). The KEGG enrichment analysis showed that 37/82 gene sets are up-regulated in phenotype SCD, 20 gene sets are significantly enriched at nominal P-value < 5%, 45/82 gene sets are downregulated in SCD, 26 gene sets are significantly enriched at nominal P-value < 5%. The KEGG result also confirmed that up-regulated gene sets in SCD were mainly associated with Lysosome (NES = 2.444, FDR q = 0.000), ECM receptor interaction (NES = 2.017, FDR q = 0.000) (Fig. 3h, i), and so on. GSEA results showed that cysteine cathepsin family members were core enriched and played an essential role in the occurrence and development of coronary heart disease.

### Protein–protein interaction network analysis and hub gene selection

**Fig. 4:**
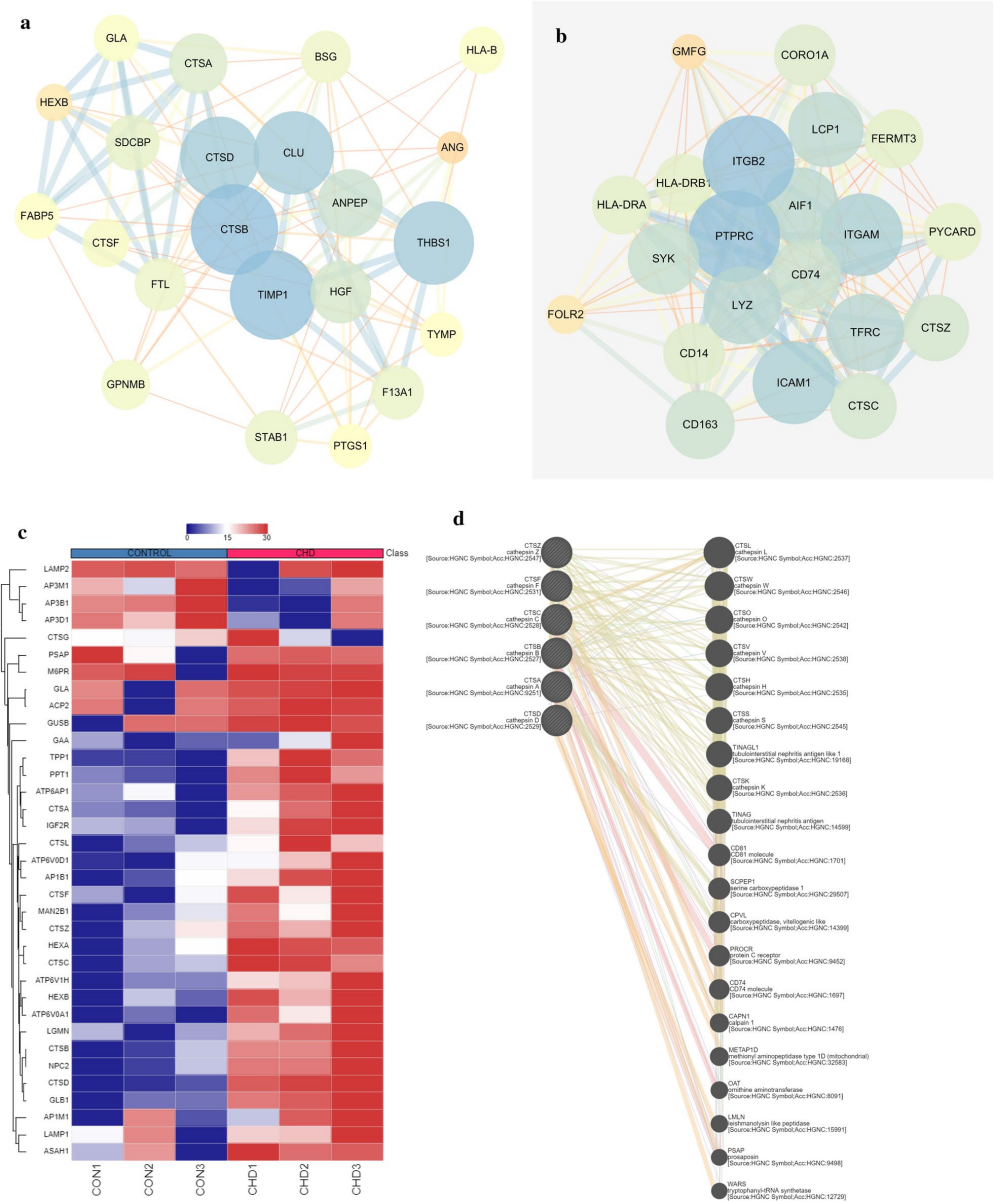
The protein–protein interaction (PPI) network, module analysis, and hub genes selection. **a**, **b** Results of subnet module analysis of the PPI network. **c** Hierarchical clustering of differentially expressed genes in lysosomal pathways, red represents up-regulated genes and staining represents down-regulated genes. **d** Gene–gene interaction network among cathepsin family members

To better understand the protein–protein relationship, we constructed the PPI network. At the same time the most significant module and hub genes of the PPI network were identified by using Cytoscape. The PPI network of DEGs consisted of 144 nodes and 392 edges, including 101 up-regulated genes and 47 down-regulated genes. According to combined score ≥ 10, two functional subnet modules are obtained from the PPI network and 43 genes were identified as hub genes from the most significant module (Fig. 4a, b). Among these genes, CTSZ, CTSC, CTSF, CTSD, CTSB, CTSA which belong to cathepsins family members were up-regulated in the SCD samples and their functional enrichment analysis were involved in lysosome pathway. Hierarchical clustering results showed that differentially expressed genes s in the lysosomal pathway could effectively distinguish SCD from atherosclerotic samples (Fig. 4c). We obtained genes -genes interaction networks among cathepsin family members via using the GENEMANIA online analysis tool (Fig. 4d). The central role of the cysteine protease family in the PPI network suggested that it played an important regulatory role in the pathogenesis of SCD.

### Verification of the expression of CTSB/ CTSC/CTSD/CTSZ

**Fig. 5:**
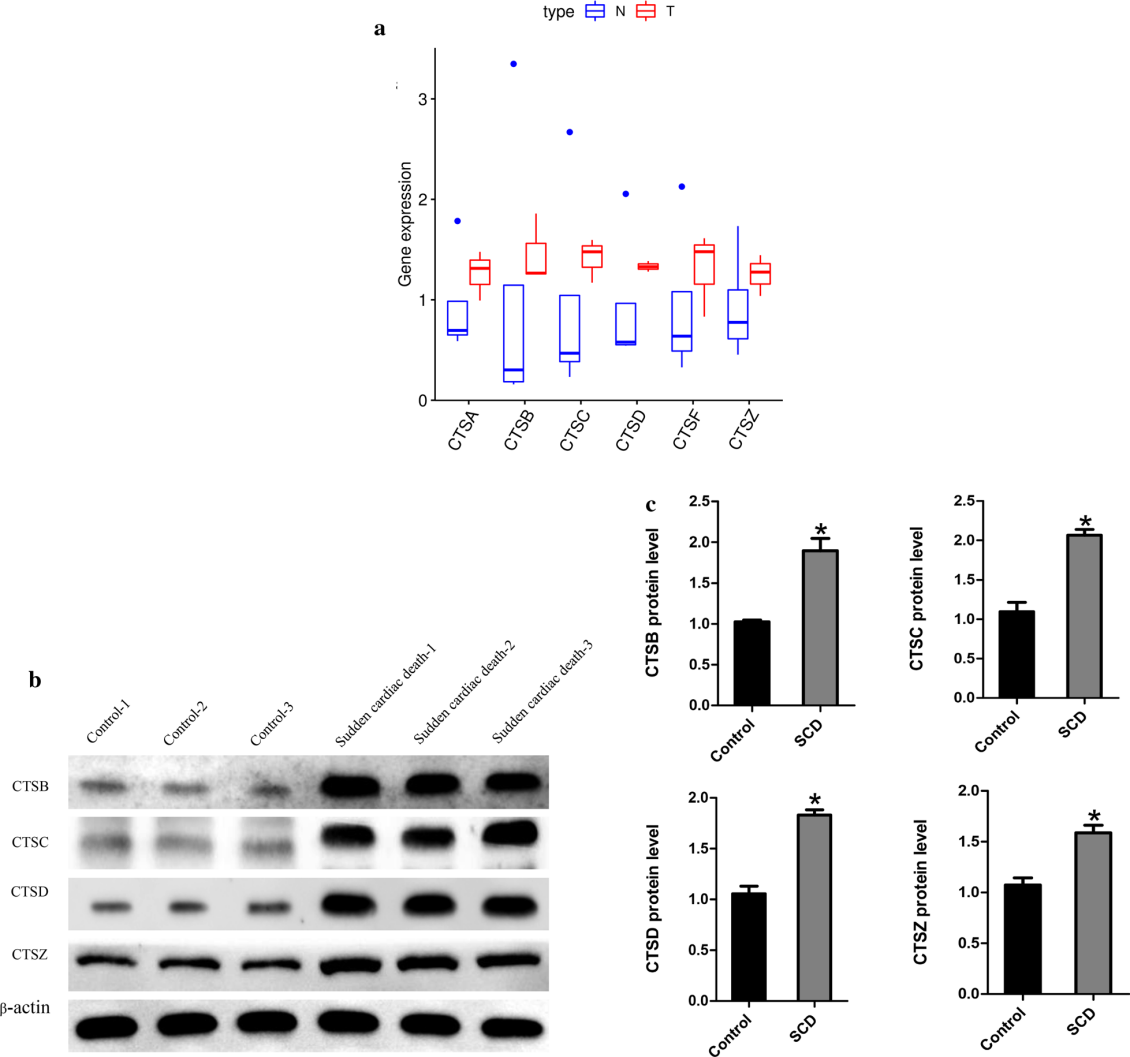
Verification of the expression of related protein. **a** Quantitative comparison of cysteine cathepsin family (CTSA/CTSB/CTSC/CTSD/CTSF/CTSZ) expression between the two groups. **b** Western blotting expression of Cysteine cathepsin family (CTSB/CTSC/CTSD/CTSZ) in the control and SCD groups. **c** Relative expression of Cysteine cathepsin family (CTSB/CTSC/CTSD/CTSZ) by Western blotting analysis. * p < 0.05, compared with control

The expression of CTSA/CTSB/ CTSC/CTSD/CTSF/CTSZ were verified via dataset and western blotting. We found these proteins were significantly up-regulated in the SCD samples in proteomics datasets (Fig. 5a). The results of Western blotting analysis displayed that the relative expression level of the SCD group was significantly higher than that of the normal group (Fig. 5b, c). The results suggested that these members of the cysteine cathepsin family may be considered biomarkers for SCD.

### The correlation between expressed cysteine cathepsin and pathological parameters of SCD

Univariate linear regression was implemented to compare expression of hub genes between different groups base on pathological parameters. The results showed that the influence of gender, BMI, Diabetes, Hyperlipoidemia, Hypertension, LVW and RVW was not statistically significant (P > 0.05). On the other hand, Gensini score was statistically significant (P < 0.05). The Degree of vascular stenosis remained related to the CTSA (R^2^ = 0.7337, P < 0.05), CTSB (R^2^ = 0.9697, P < 0.05), CTSD (R^2^ = 0.8094, P < 0.05), CTSF (R^2^ = 0.8359, P < 0.05), CTSZ (R^2^ = 0.8244, P < 0.05) in the univariate linear regression mode (Table 1). It was shown that the expression of cysteine cathepsin in coronary arteries was positively correlated with the severity of CHD.

**Table 1:** The linear regression analysis between SCD and relevant genes expression

Gene symbol	Gensini score	
	Univariate linear regression	
	R square	P value
CTSD	0.7337	0.0294*
CTSB	0.9697	0.0003*
CTSF	0.8094	0.0146*
CTSZ	0.8359	0.0107*
CTSA	08,244	0.0123*
GLA	0.8690	0.0067*
TYMP	0.8977	0.0041*
FTL	0.9701	0.0003*
PTGS1	0.9726	0.0003*
HEXB	0.8437	0.0097*
TIMP1	0.8502	0.0089*
THBS1	0.7861	0.0186*
CD14	0.6848	0.0420*
AIF1	0.8708	0.0066*
PTPRC	0.8762	0.0060*
ITGB2	0.9809	0.0001*
ITGAM	0.8915	0.0046*
FERMT3	0.6938	0.0395*
CD74	0.7708	0.0214*
PYCARD	0.7593	0.0238*
TFRC	0.7913	0.0176*

*Screening and correlation analysis of autophagy related* gene.

**Fig. 6:**
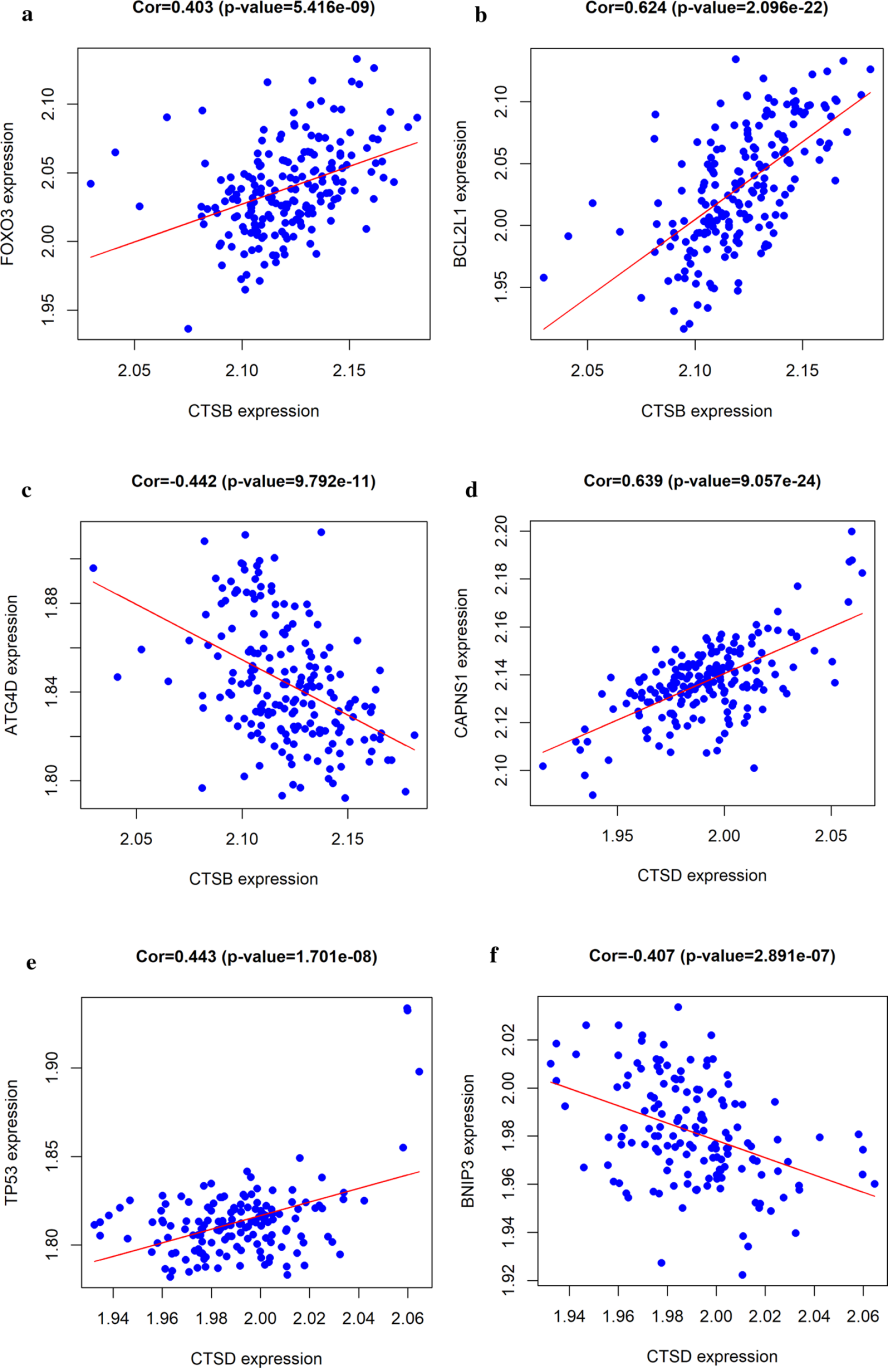
Correlation between CTSB/CTSD and autophagy-related genes. **a**, b CTSB expression was positively correlated with **a** FOXO3, **b** BCL2L1, but negatively correlated with **c** ATG4D. CTSD expression was positively correlated with (D) CAPNS1, (E) TP53, but negatively correlated with (F) BNIP3

The role of autophagy in ischemic heart disease may be complex and can be activated when coronary artery stenosis occurs. CTSB and CTSD were extracted as the autophagy-related genes from Human Autophagy Database(HAD). In addition, we analyzed the relationship between these two autophagy related genes with other autophagy genes of two other datasets (GSE12288, GSE20680), the result of the correlation between CTSB/CTSD and autophagy-related genes showed that CTSB genes expression was negatively correlated with ATG4D (P < 0.001, r = 0.41; Fig. 6c), but positively correlated with FOXO3 (P < 0.001, r = 0.44; Fig. 6a), and BCL2L1 (P < 0.001, r = 0.63; Fig. 6b). CSTD expression, on the other hand, was negatively correlated with BNIP3 (P < 0.001, r = 0.41; Fig. 6f), but positively correlated with CAPNS1 (P < 0.001, r = 0.65; Fig. 6d), and TP53 (P < 0.001, r = 0.45; Fig. 6e). The result can be speculated that CTSD/CTSB regulates SCD through autophagy.

### Analysis of TF-mRNA-miRNA regulating networks

**Fig. 7:**
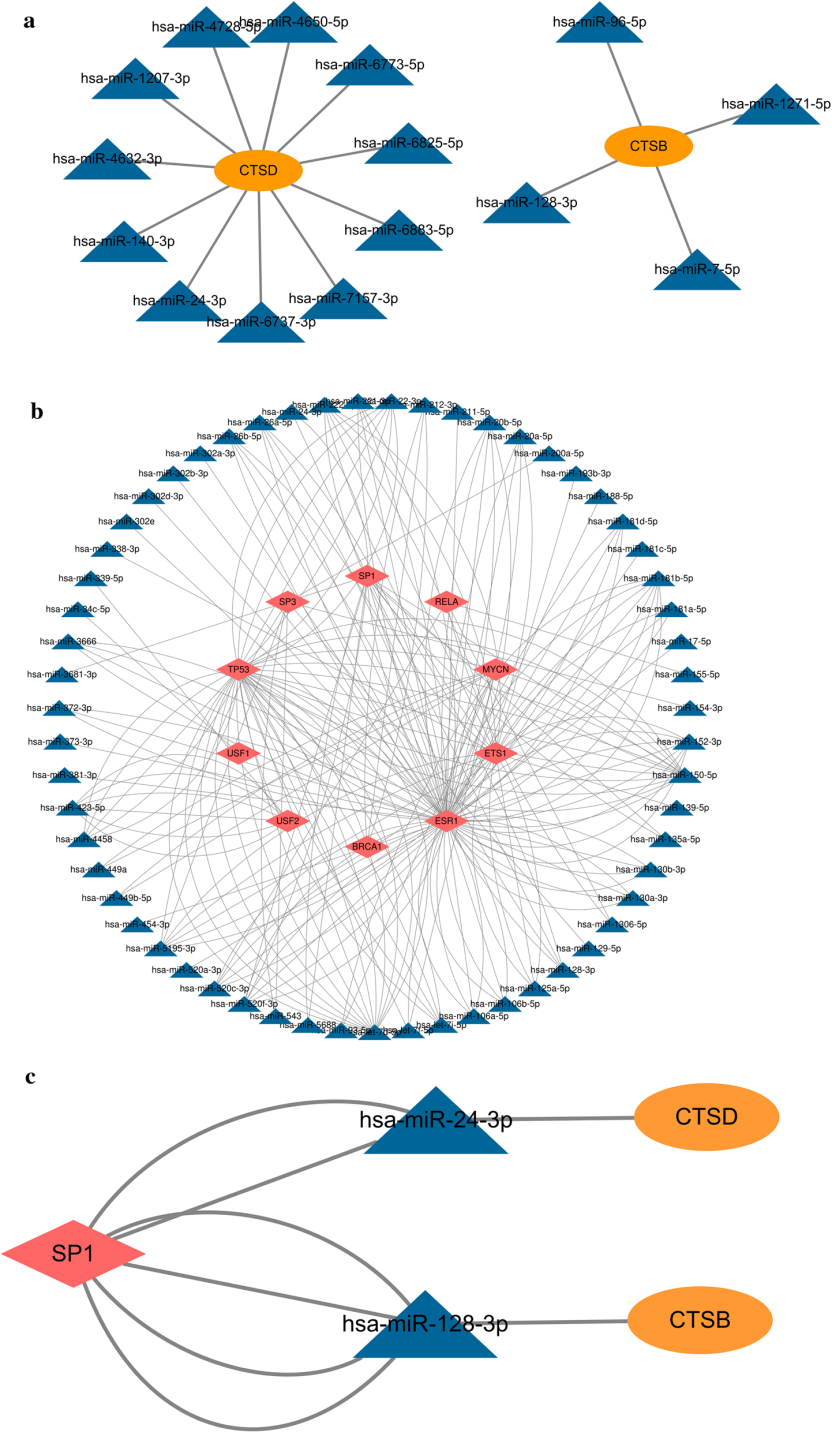
The TF-miRNA-mRNA regulatory networks were constructed. (**a**) mRNA-miRNA regulatory network. The diamond represents miRNA, and the green circle represents CTSB/CTSD. (**b**) TF-miRNA regulatory network. (**c**) TF-miRNA-mRNA regulatory network. The blue triangle represents miRNA, the red diamond represents TF and orange circle represent CTSB/CTSD

The regulated networks have been recognized that it plays a crucial role in understanding the mechanism of disease, so we predicted the interactions of miRNA-mRNA and TF-mRNA with Networkanalyst online tools. After that, a TF-mRNA-miRNA triple network was constructed. In the result of in the miRNA-mRNA regulatory network, including 2 DEGs nodes and 15 miRNA nodes (Fig. 7a). In the TF-mRNA regulatory network, including 2 DEGs nodes and 194miRNA nodes (Fig. 7b). In triple network, the regulatory relationships between miRNA, TF, mRNA regulating networks and up-regulated cysteine cathepsin family genes were found, SP1-CTSD-(has-miR-24-3p) and SP1-CTSB-(has-miR-128-3p) from the TF-mRNA-miRNA network were identified as playing a potentially critical regulatory role in CHD (Fig. 7c).

## Discussion

SCD is a significant public health issue, which accounts for half of all cardiovascular-related deaths worldwide [2, 23, 24]. In autopsies performed on adult male who are victims of sudden cardiac death, we found that these deceased had atherosclerotic plaque led to various degrees of coronary artery stenoses, which resulted in the dysfunction of cardiomyocytes and cardiac functions.

To investigate the role of autophagy in SCD, we analyzed the datasets which performed protein quantification of the anterior descending branch of the left coronary artery in six cadavers (SCD = 3, control = 3) using label-free proteomics. we identified 148 DEPs, and we screened out 6 members of the cathepsins family (CTSZ, CTSC, CTSF, CTSD, CTSB, CTSA) as hub proteins through Functional enrichment, PPI module analysis. The relative expression level was significantly higher in the CHD group compared with the normal group, and their functional enrichment analysis were involved in inflammation, immune response, and lysosome pathway, the same results were still obtained with Metascape, GSEA and Hierarchical clustering. We focused on autophagy-associated cathepsins in cathepsins family which were obtained by comparing the human autophagy database so as to analyze the expression levels of expressed, regulatory networks, and potential mechanism of CTSB/CTSD in SCD.

Cathepsins are the major lysosomal hydrolases [25]. Their activities potentially affect crucial atherogenic cascades, such as the inflammation, lipid metabolism, and autophagy [26]. Previous clinical studies suggested that CTSB and CTSD were known as plays a crucial role in arterial stiffening and atherosclerotic vascular disease [23, 27]. The serum levels all associated with increased risk and poorer outcome of coronary events severity [28–31]. Above conclusion was also confirmed via the quantification of proteomics and western blotting in our study. In addition, we also found that the expression of CTSB/CTSB in SCD were positively correlated with the Gensini score (which is a well-recognized scoring system that evaluate the severity of CHD [32, 33]). Yet, a few studies have a contrasting conclusion, they reported that decreased myocardial and serum CTSD levels were relatively lower in SCD with cardiac hypertrophy [34]. The different conclusion may be related to a number of factors (e.g., the basal diseases, source of samples, sample collection time et al.). Despite the opposite conclusion, to be sure, CTSB/CTSD played an important role in the development of SCD and involved the severity of atherosclerosis.

It is increasingly appreciated that autophagy can be both protective and deleterious in atherosclerotic. Under the ischemic, hypoxia and hyperlipidemia stress, in order to deal with the cytotoxic effects of excessive inflammation, autophagy is activated to protect cardiomyocytes against ischemic or hypoxia injury and inhibition of inflammation, so as to contributes to enhances atherosclerotic plaque stability and attenuate cardiac injury. As an indicator of autophagic activity, CTSB and CTSD were involved in the regulation of cell death and survival in the development of atherosclerosis [35]. Si Ming Mand et al. believed that under homeostatic conditions CTSB cleaves the calcium channel MCOLN1/TRPML1 in the lysosomes, maintaining suppression of TFEB and reducing expression of lysosomal and autophagy-related proteins [36]. As another indicator of autophagic activity. Cardiac autophagy activity was increased after myocardial infarction, if up-regulation of CTSD was prevented during myocardial infarction exacerbates poor cardiac remodeling and dysfunction in mice [31]. Some studies had also recognized that after the silencing of cathepsin D, apoptosis and necrosis significantly increased, while stress-induced autophagy was abrogated [37]. From the above viewpoints, upregulation of CTSB/CTSD by atherosclerosis promotes autophagic flux and protects against cardiac remodeling and heart failure. Based on the above, CTSB/CTSD can be considered as a potential biomarker.

Besides its protective activities, more and more evidence showed that dysfunction of autophagy is an essential contributor to the development of advanced atherosclerotic lesions [38],it perhaps play a detrimental role in plaque formation, excessive or uncontrolled levels of autophagy are able to induce autophagy-dependent cell death and promoted atherosclerotic plaque instability [4]. Our results prefer to this conclusion. In our analysis, overexpression of CTSB and CTSD in SCD, we considered that CTSB/CTSD was involved in mechanisms of early atherogenesis and activated an autophagic response, but from the advanced atherosclerosis stage to SCD, the cholesterol crystals in the continuous formation of plaque destroy the lysosomal membrane, disrupt the autophagy process [39],and autophagy promotes atherosclerosis through of excessive inflammasome activation [13],the other is persistent hypoxic or ischemia, autophagy fails to deal with the excessive amount of oxidative stress in the plaque, finally lead to cell death, i.e., apoptosis. However, the two different perspectives indicating the controversial effect of autophagy in atherosclerosis need more thorough research.

In order to further explore regulatory mechanisms of CTSB/CTSD in autophagy, we performed a correlation analysis of CTSD/CTSB with other autophagy-related genes. The results obtained in this study showed that the expression of CTSB was negatively correlated with ATG4D, but positively correlated with FOXO3, the expression of CSTD, was negatively correlated with BNIP3, but positively correlated with TP53.However, the results of our analysis do not fit the trend of BNIP3 and ATG4D in autophagy in the literature [40–43],The specific reasons and mechanism merit further exploration.

Although changes in CTSD/CTSB affect the occurrence of CHD, the main causes and underlying mechanisms are unclear. In this study, to better understand the mechanism of CTSD/CTSB in sudden coronary heart disease death, we also analyzed the TF-mRNA-miRNA relationship to obtain the co-regulatory network. SP1-CTSD-(has-miR-24-3p) and SP1-CTSB-(has-miR-128-3p) from the TF-mRNA-miRNA network were identified to play crucial roles in CHD. SP1 is a zinc finger transcription factor that regulates target gene transcription by binding to their promoter contain GC boxes, including regulate the expression of cathepsin [44]. It is also associated with several cellular processes such as chromatin remodeling, cell growth, responses to DNA damage, apoptosis, and cell differentiation. Experimental studies have revealed an essential role for microRNAs in regulating molecular and cellular processes related to the development of atherosclerosis. has-mir-24-3p is associated with plaque progression and plaque instability [45], while has-mir-128-3p is a key regulator of VSMC, affecting proliferation, migration, differentiation, and contraction of VSMC [46]. Thus, in the present study, we gain further insight into the mechanisms of target genes in the disease from the regulatory.

## Conclusion

In our present study, we used a proteomic dataset of the left anterior descending branch of the human coronary artery from sudden cardiac death due to coronary heart disease, and screening the DEGs to focus on the cathepsin family members. The expression and function of the gene family members in CHD were comprehensively analyzed, and the CTSD/CTSB associated with autophagy were selected for mechanism study. Moreover, we have to admit that our study had limitations, although the use of human specimens avoids the drawbacks of tissue specificity, and the experimental results were more convincing for the development of the disease, but the sample size was small and only the male gender was analyzed. It may have had some influence on the results, so in the next step, it requires us to take into account plausibility to design the dataset. we will perform the relation between Cathepsins and autophagy by in vitro study as well by using western blot, immunofluorescence, immunoprecipitation. In summary, our findings provide new insights into the pathogenesis of CTSB/CTSD in SCD.

## Data Availability

The datasets used and analyzed during the present study are available from the corresponding author on reasonable request.

## Abbreviations

AMPK: Adenosine 5 ‘-monophosphate (AMP)-activated protein kinase
APP: Amyloid precursor protein secretase
ATG: Autophagy related genes
ATG16L1: Autophagy related 16 Like 1
ATG4D: Autophagy related 4D cysteine peptidase
BAG1: BAG cochaperone 1
Bcl2: BCL2 apoptosis regulator
BCL2L1: BCL2 Like 1
BMI: Body mass index
BNIP3: BCL2 interacting protein 3
BP: Biological processes
CAD: Coronary artery disease
CAMKK2: Calcium/calmodulin dependent protein kinase kinase 2
CAPNS1: Calpain small subunit 1
CC: Cell components
CHD: Coronary heart disease
CTSA: Cathepsin A
CTSB: Cathepsin B
CTSC: Cathepsin C
CTSD: Cathepsin D
CTSF: Cathepsin F
CTSZ: Cathepsin Z
DAVID: Database for aAnnotation, visualization and integrated discovery
DEG: Differentially expressed gene
ECM: Extracellular matrix
FC: Fold change
FDR: False discovery rate
FOXO3: Forkhead box O3
GEO: Gene expression omnibus database
GO: Gene onotology
GSEA: Gene set enrichment analysis
HRP: Horseradish peroxidase
ICM: Ischemic cardiomyopathy
ITGA6: Integrin subunit alpha 6
KEGG: Kyoto encyclopedia of genes and genomes
LVW: Left ventricular wall
MCODE: Molecular complex detection
MF: Molecular function
MI: Myocardial infarction
miRNA: MicroRNA
mRNA: Messenger RNA
mTOR: Ammalian target of rapamycin
PCA: Principal component analysis
PPI: Protein–protein interaction
PVDF: Polyvinylidene fluoride
ROS: Reactive oxygen species
RVW: Right ventricular wall
SCD: Sudden cardiac death
SDS-PAGE: Sulfate polyacrylamide gel electrophoresis
SP1: Sp1 transcription factor
TF: Transcription factors
TFEB: Transcription factor EB
TP53: Tumor protein p53
ULK1: Unc-51 like autophagy activating kinase
VSMC: Vascular smooth muscle cells
